# Bioconversion efficiency and chemical composition of *Hermetia illucens* larvae fed spent mushroom substrates

**DOI:** 10.1186/s13568-024-01802-4

**Published:** 2024-12-13

**Authors:** Anjani Nayak, Martin Rühl, Patrick Klüber

**Affiliations:** 1 Institute of Food Chemistry and Food Biotechnology, Justus Liebig University Giessen, Giessen, Germany; 2 Fraunhofer Institute for Molecular Biology and Applied Ecology, Ohlebergsweg 12, 35392 Giessen, Germany

**Keywords:** Bioconversion, Black soldier fly, Circular economy, Agricultural by-products, Insect rearing, Spent mushroom substrate

## Abstract

Spent mushroom substrate (SMS) is a by-product remaining after harvesting mushrooms. We evaluated the effect of substituting chicken feed with 0–100% of *Pleurotus eryngii* and *Lentinula edodes* SMS at different stocking densities (200–1000 larvae/box) on development, composition, and substrate reduction of black soldier fly (*Hermetia illucens*) larvae. Although the survival rate was not significantly different, feeding pure SMS led to a low growth rate. The substitution level of SMS negatively correlated with individual larval weight, total harvested biomass, larval growth rate (LGR), feed conversion ratio (FCR), substrate reduction, and waste reduction index (WRI) except for the 20% substitution. Feeding 40% SMS resulted in the highest number of prepupae. In the density experiment, the heaviest larvae (220–239 mg fresh weight) were obtained at 200 larvae/box in the 0% SMS group. The frass residue and FCR decreased with increased density. Remarkably, when feeding 20% SMS at 250 larvae/box, the harvested biomass, LGR, and FCR did not differ significantly from the 0% SMS control, whereas some of the higher densities led to a deterioration. In fact, the frass residue, substrate reduction, and WRI were even improved at 250 larvae/box in the 20% SMS group. The chemical analyses of larvae reared on 20% SMS at 250 larvae/box showed comparable ash and fat contents and a higher protein content compared to the 0% SMS group. Accordingly, up to 20% of a standard diet such as chicken feed can be replaced by low-cost SMS without disadvantages for breeding.

## Introduction

The expected global increase in demand for animal protein is 14% per person in the next 30 years (Komarek et al. 2021). The livestock-production, processing, and transport do not only consume valuable resources but are responsible for up to 45% of the greenhouse gas emissions (Gerber et al. 2013). As a consequence of the growing demand for animal protein, the demand for feed is also steadily increasing. The quantity of cereals used in animal feed is estimated to reach over 1.1 billion tons by 2050 (Makkar 2018). It is also changing land use patterns, forcing the destruction of natural ecosystems such as (rain)forests across the world. Therefore, the exploration of sustainable alternatives, such as agricultural by-products, to commercially used feed is urgently needed. However, the digestion and absorption of certain by-products by conventional livestock may be limiting because of the presence of anti-nutritional factors (Samtiya et al. 2020) and contaminants (Jayathilake et al. 2022). Since insects have the capability to feed on various organic matter, they are considered promising bioagents that could be part of an effective waste management in the future, thus contributing to the circular economy (Boukid et al. 2021). Such insects, which are rich in their nutritional composition, can in turn be fed to animals. Thus, reducing the dependability on importing feed ingredients.

Larvae of the black soldier fly, *Hermetia illucens* (BSF; Diptera: Stratiomyidae), are well known for their remarkable capability in thriving on diverse biodegradable materials. It is hence identified as a potential candidate for converting agricultural by-products into nutrient-rich biomass (Siddiqui et al. 2022). This conversion process also produces frass, a mixture of insect exuviae, remaining feed and feces; which can be used as a soil supplement (Klammsteiner et al. 2020). The BSF is native to South America and requires warm and humid climatic conditions (Čičková et al. 2015). They are polyphagous insects with a voracious feeding appetite at their larval stage. The life cycle under optimum conditions is around 45 days (De Smet et al. 2018). The larval stage consisting of six instars covers 14–16 days in ideal conditions (Tomberlin et al. 2002, 2009). There are several studies on the ability of BSF larvae in bioconverting by-products including restaurant wastes (Zheng et al. 2012a, b; Leong and Kutty 2020; Nguyen et al. 2013), fish offal (St-Hilaire et al. 2007), beer production wastes (Chia et al. 2018), human and livestock feces (Sheppard et al. 1994; Newton et al. 2005; Myers et al. 2008; Rehman et al. 2017), and municipal organic wastes (Kalová and Borkovcová 2013) to nutrient-rich feed alternatives. Despite the promising and versatile application possibilities of BSF larvae in waste management, feeding of insects is regulated within the legislative framework of the European Union (EU). Since industrially reared insects are classified as “farmed animals”, the same general rules and restrictions apply as for conventional livestock. Thus, farmed insects are subject to the feed ban rules laid down in Article 7 and Annex IV to Regulation (EC) No 999/2001 and, additionally, to animal feeding rules laid down in Regulation (EC) No 1069/2009. According to the abovementioned regulations, insects are not allowed to be fed with slaughterhouse or rendering-derived materials, feces, catering waste, and unsold supermarket or industrial products that are containing fish or meat (Veldkamp et al. 2022). On one hand, it constricts the available options to feed the insects sustainably without depending on importing feed grains. On the other hand, it provides a gateway to look at the other available plant-based by-products as feed for the larvae. Unfortunately, there are plenty of agricultural by-products ending up in landfills or are burned. Besides losing valuable resources there is also pollution caused by the way these wastes are currently handled (Nguyen et al. 2015). Therefore, identifying the by-products and utilizing them as an alternative feed source would solve multiple problems. This way the competition between the resources for food and feed production can also be minimized.

Among all the food products with emerging demand, mushrooms being healthy and rich in protein are particularly attractive. According to Market Data Forecast (2022), the European mushroom industry (consisting mainly of button, shiitake, and oyster mushrooms) had a market value of USD 13.7 billion in 2018 and is estimated to reach USD 21.7 billion by 2026. Mushroom cultivation includes a substrate on which the mycelium grows. The substrate consists of lignocellulose-rich wastes like wood logs, rice straw, horse and chicken manure, wheat straw, wood chips, sawdust, seed husks, coffee pulp, corn cobs, and bagasse among many others (Oei 2003). To ensure an optimal milieu for mycelial growth and fruiting body formation, substrate properties and processing steps must be adjusted to the requirements of the corresponding mushroom. Once the mushrooms have been harvested, the by-product remaining is the growing substrate, also known as spent mushroom substrate (SMS). Astonishingly, the production of 1 kg of mushrooms generates 3–5 kg of waste (Beckers et al. 2019; Leong et al. 2022). The amount of SMS waste generated in the EU alone is more than 3 billion kilograms per year (Ceurstemont 2020). This tremendous amount of waste has to be managed and mostly ends up in landfills, costing 10–50 € per ton (Beckers et al. 2019). SMS is already being examined for its suitability as fertilizer, alternative feed source, in pest management (Rinker 2017), for potential reuse in mushroom cultivation (Rinker 2017; Zied et al. 2020), and in ethanol production (Grover et al. 2015). Although there are several concepts to prevent SMS from being dumped in landfills, these solutions are not applied in practice. Another barely studied option is to utilize SMS as insect feed. In this way, the extensively produced and locally available by-product of the mushroom industry can be upcycled into high-value insect protein and fat within a short time.

The BSF larvae could become a biological tool in reducing the amount of SMS that is otherwise discarded. Encompassing all publications on feed trials with BSF larvae, there are only two studies so far highlighting the use of SMS (Li et al. 2021; Mao et al. 2023). In the former study, the SMS was mixed with canteen waste. However, the use of food waste from restaurants as larval feed is not allowed under current EU regulations. Commercial BSF producers predominantly use chicken or pig feed as diet for larval fattening. The high costs of the feed and imported grains make it expensive and unsustainable, resulting in producers having difficulty competing with conventional animal and plant protein sources. The use of SMS as a complete substitute or, depending on the growth performance, as a feed supplement could therefore considerably reduce the costs of insect production.

The initial experiment focused on the ability of BSF larvae to survive on diets consisting of chicken feed replaced with different ratios of SMS. Subsequently, we aimed to find the ideal stocking density (200–1000 individuals) per 100 g feed to obtain the optimum survivability, growth performance, feed conversion efficiency, and waste reduction. Additionally, the larval nutritional composition was analysed to evaluate the potential of BSF larvae as novel feed.

## Materials and methods

The experiments were conducted between July 2021 and June 2022 at the Fraunhofer Institute for Molecular Biology and Applied Ecology (Giessen, Germany).

### Insects and feeding substrates

Eight-day-old BSF larvae were shipped vacuum-packed by Hermetia Baruth GmbH (Baruth/Mark, Germany) and used for the feeding trials. After receiving the larvae, they were unpacked and transferred into polypropylene boxes and kept for 1 h in a climate chamber at 27 ± 1 °C and 65 ± 5% relative humidity to recover natural behavior. To ensure uniform size and mass they were separated using a vibratory sieve shaker with a mesh size between 1.0 and 1.4 mm (AS 200, Retsch, Haan, Germany). The mean individual larval weight was determined by weighing five replications of 100 larvae with a precision balance (ALJ 160-4 A, Kern & Sohn, Balingen-Frommern, Germany). BSF larvae that were used for feeding trials had an average individual weight of 6.0 ± 1.2 mg.

The diets tested consisted of chicken feed (CF; GoldDott Eierglück, DERBY Spezialfutter, Muenster, Germany), SMS, or combinations thereof, with CF serving as a high-quality reference diet (Klüber et al. 2023). The SMS was obtained from an organic mushroom farm (Löckes Bio-Vertriebs GmbH, Büttelborn, Germany). For this, the SMS was collected as a by-product of king oyster (*Pleurotus eryngii*) and shiitake (*Lentinula edodes*) production on the same day of harvesting the fruiting bodies. First, remaining fruiting bodies attached to the surface of the SMS blocks were removed. Subsequently, the blocks were disintegrated by hand and dried immediately at 80 °C for 10 h in a laboratory kitchen oven (HB674GBS1, Siemens AG, Munich, Germany). The CF and dried SMS were prepared by grinding in a Mockmill 200 (Wolfgang Mock, Otzberg, Germany) and a Thermomix TM6 (Vorwerk, Wuppertal, Germany) to a particle size of 0.1–0.8 mm. The substrates were stored in airtight containers at room temperature until use. King oyster SMS and shiitake SMS were mixed in a 1:1 ratio for the feeding trials and are summarized hereafter as SMS.

### Gradual substitution of standard feed with SMS

First, it was intended to clarify how the growth performance and survivability of larvae are affected if different proportions of CF are replaced by SMS. A total of six substitution levels were examined (0%, 20%, 40%, 60%, 80%, 100%), with 0% substitution representing the pure CF control diet.

We weighed 150 g (dry matter; DM) of each diet into four conical 12.5 × 12.5 × 11.5 cm (l × w × h) replicate boxes (BDPN24, MegaView Science, Taichung, Taiwan) and adjusted the moisture to 60% by adding warm tap water. Subsequently, 200 eight-day-old BSF larvae were transferred in each box by spreading them over the substrate surface. The donut-shaped lid was fitted with a circular 9 cm mesh insert for a proper air circulation. All boxes were incubated in a climate chamber under controlled conditions of 27 ± 1 °C and 65 ± 5% relative humidity in darkness. The boxes were rearranged randomly on every second day. No additional feed or water was added throughout the experiment. Based on the findings of preliminary experiments, larvae of all dietary groups were harvested ten days after the start of feeding (first prepupae observed). For this purpose, larvae were collected individually from the frass using spring steel tweezers, cleaned of coarse impurities, weighed, counted, and cold-inactivated at − 20 °C. The survival rate was determined as the percentage of larvae recorded during harvesting. The total harvested biomass represents the total amount of harvested insect biomass per box (larvae, prepupae, and pupae). On this basis, individual larval weight was calculated by dividing the total harvested biomass by the number of surviving larvae (given as fresh matter; FM). The frass residue is defined as the amount of digested substrate remaining after harvesting the larvae. The weight of the frass was obtained by differential weighing with the empty box. After mixing and homogenization, the moisture content was determined (DAB 100-3, Kern & Sohn, Balingen-Frommern, Germany) to reveal the dry weight of the frass residue. For the calculation of conversion efficiencies and substrate reductions it was assumed that the entire feed provided had been consumed by the BSF larvae (Oonincx et al. 2015). All data were recorded in four biological replicates. Besides larval growth rate (Eq. 1), the following parameters depicted in Eqs. (2–5) were calculated based on DM (Oonincx et al. 2015; Mohd-Noor et al. 2017; Jucker et al. 2020):

1 $$\begin{aligned}&LGR \left(Larval \; growth \; rate\; (g/d)\right)\\ &\; = \frac{(Total \; biomass \; harvested \; (g)-Initial\; biomass\; inoculated\; (g))}{Number \; of \; rearing \; days\; (d)}\end{aligned}$$

2 $$\begin{aligned}&FCR \left(Feed\; conversion\; ratio\right)\\ &\;= \frac{Total\; feed\; provided\; (g)}{(Total\; biomass\; harvested\; (g)-Initial\; biomass\; inoculated\; (g))}\end{aligned}$$

3 $$\begin{aligned}&ECI \left(Efficiency\; of\; conversion\; of\; the\; ingested\; feed\right)\\ &\;=\frac{(Total\; biomass \; harvested \; (g)-Initial \; biomass \; inoculated \; (g))}{(Total \; feed \; provided \; \left(g\right)-Frass \; residue \; \left(g\right))}\end{aligned}$$

4 $$\begin{aligned}&SR (Substrate \; reduction\; (\%))\\ &\;= \frac{\left(Total \; feed \; provided \; \left(g\right)-Frass\; residue\; \left(g\right)\right)\times 100}{Total\; feed \; provided \; (g)}\end{aligned}$$

5 $$\begin{aligned}&WRI \left(Waste\; reduction\; index\right)\\ &\;= \frac{(Total\; feed\; provided\; \left(g\right)-Frass\; residue\; \left(g\right))}{Number\; of\; rearing\; days\; (d)}\end{aligned}$$

### Optimization of stocking density

After demonstrating that BSF larvae were able to survive on substrate substituted with SMS, we aimed to identify the best combination of stocking density and SMS substitution level at which larvae grow best while exhibiting high conversion efficiency and substrate reduction. Since larval growth in the gradual substitution experiment was strongly inhibited when the SMS level was ≥ 60%, chicken feed was replaced by 50% or less to find density-related effects. Feed, frass and larval samples were stored at − 20 °C until further use.

A total of four different density populations (200, 250, 400, 1000 larvae/box) were combined with four SMS substitution levels (0%, 20%, 40%, 50%). The feeding trials were conducted under identical conditions as described in the gradual substitution experiment, with only 100 g of feed being provided. All data were recorded in four biological replicates, besides the 400 larvae/box approach which was replicated three times. The corresponding data collection and calculations were also performed according to the same methodology mentioned above.

### Chemical analysis

The highest individual weight of larvae was found at a density of 250 larvae/box within the SMS containing groups. Moreover, when feeding 20% SMS at 250 larvae/box, the harvested biomass, LGR and FCR did not differ significantly from the 0% SMS control, whereas some of the higher densities led to a decrease. In fact, the frass residue, substrate reduction and WRI were even improved at 250 larvae/box in the 20% SMS group. Therefore, the feed, larvae, and frass were examined for their nutritional composition at a density of 250 larvae/box. Among the harvested biomass, prepupae and pupae were not included in the chemical analyses. All chemical analyses were conducted in triplicates except pH measurements which were done in duplicates.

All parameters studied refer to DM (except dry matter content). Feed (~ 150 g), L5 larvae (~ 100 g), and frass (~ 200 g) of the four replicates within a group were quantitatively transferred to a box and pooled. The samples were then ground with a mortar under liquid nitrogen and lyophilized at 0.8 mbar vacuum pressure and − 85 °C condenser temperature (Delta LSCplus, Martin Christ Gefriertrocknungsanlagen, Osterode am Harz, Germany) for 72 h. Prior to lyophilization, the moisture content was determined thermogravimetrically (M35 Moisture Analyzer, Sartorius, Göttingen, Germany). For the analyses of crude ash, protein, and fat, 1 g of lyophilized feed and frass, as well as 0.5 g of lyophilized larvae were used. However, 3 g of the samples was used to determine crude fiber.

The initial pH value of the feed and the pH value of the frass after harvesting were measured (S47-K, Mettler-Toledo, Gießen, Germany). For this, 5 g of the sample was added to 40 mL distilled water, mixed thoroughly and centrifuged to measure the pH of the supernatant. The crude fiber content was determined according to the method of Scharrer-Kürschner, as described elsewhere (Matissek et al. 2018). The crude ash was measured by pre-ashing the samples over a Bunsen burner and incinerating them twice for 6 h at 550 °C in a muffle furnace (L 9/11, Nabertherm, Lilienthal, Germany). The content was then calculated by differential weighing. The determination of total nitrogen was conducted according to the method of Kjeldahl (Matissek et al. 2010), where the samples were subjected to digestion in concentrated sulfuric acid, followed by steam distillation (behrotest S5, behr Labor-Technik, Düsseldorf, Germany) and titration (TitroLine 5000, SI Analytics, Mainz, Germany). We used conversion factors based on the corresponding amino acid profile to calculate the crude protein content. Hydrolysis of samples (20–40 mg) for amino acid profiling was achieved using three different protocols. The total hydrolysis covers all amino acids except tryptophan, cysteine and methionine. Consequently, alkaline and oxidative digestion were performed for tryptophan and both sulfur-containing amino acids, respectively (Seidel et al. 2023). The released amino acids were then separated by a cation exchange column, derivatized with ninhydrin and detected at 570 nm–440 nm (proline) in the amino acid analyzer (S433, Sykam Chromatographie Vertriebs GmbH, Fürstenfeldbruck, Germany). To determine the crude fat content according to Weibull-Stoldt, the samples were disintegrated in boiling 4 mol⋅L^–1^ hydrochloric acid for 30 min. Thereafter, samples were filtered, washed neutrally with hot demineralized water, and dried to constant weight for ~ 1 h at 105 °C. The extraction of the fat was performed in a behr E6 system using petroleum ether (behr Labor-Technik, Düsseldorf, Germany) and the content was determined gravimetrically. The extracted lipids were dissolved in 3 mL of iso-octane and stored at − 20 °C. According to the method of Hammer et al. (2021), fatty acids (FAs) bound in triglycerides were converted into fatty acid methyl esters (FAMEs) by means of alkali-catalyzed transmethylation. The generated FAMEs were separated by gas chromatography (7890B, Agilent, Waldbronn, Germany) using an Agilent VF-WAXms column (30 m × 0.25 mm, 0.25 μm film thickness) with 1.56 mL/min as carrier gas flow. After the column, the gas flow was split into two halves. One led to an Agilent 5977B MSD detector, while the other half led to an olfactory detection port that was not used for FA analysis. The temperature program and detector parameters have been described elsewhere (Hammer et al. 2021). The 37 Component FAME Mix (Supelco, Bellefonte, Pennsylvania, USA) was used to identify the FAMEs.

### Data processing and statistics

Data curation and processing were carried out using Excel 2016 (Microsoft, Redmond, WA, USA). Statistical analysis and visualization were conducted in OriginPro 2022b (OriginLab, Northampton, MA, USA). The Shapiro–Wilk test was applied to verify whether the data of a population is normally distributed. The homogeneity of variance was calculated with Levene’s test. Parameters recorded in the gradual substitution experiments and chemical analyses were subjected to a one-way analysis of variance (ANOVA) and means were separated using the Tukey’s test (homogeneous variance). If the variance was inhomogeneous, a one-way Welch’s ANOVA followed by Games–Howell post hoc test were conducted. The Pearson product–moment correlation was used for determining linear relationships between variables (Hilgers et al. 2019). Data obtained from the stocking density experiments were subjected to a two-way ANOVA and means were separated using the Tukey’s test. An error level of *α* = 0.05 for statistical significance was set for all analyses.

## Results

### Feeding gradual substitutions of SMS

First, the extent to which a standard feed can be substituted by SMS was examined in order to achieve identical or improved growth performance and biomass yield. All groups received exactly the same amount of 150 g DM of the corresponding diet, with high-quality CF being gradually replaced by 0–100%. The survival rate of pure CF and all substitution groups did not differ significantly, whereas the 40–80% SMS groups were found to have ≥ 3% higher survival rates compared to pure SMS (*F*_5,18_ = 4.84; *P* ≤ 0.04; Table 1). There was no linear correlation between the survival rate and the SMS level (*r* = − 0.14; *P* = 0.51). The highest individual larval weight (223–234 mg FM) was obtained in the 20% SMS group and was in fact 11.5% greater than the CF larvae (*F*_5,18_ = 1012.06; *P* < 0.00009). Contrastingly, individual larval weight was strongly negatively correlated with increasing SMS level (*r* = − 0.96; *P* < 0.00001) and decreased drastically by up to 93.2% and 93.9% when compared to the CF and 20% SMS groups, respectively (*F*_5,18_ = 1012.06; *P* < 0.00001).

**Table 1:** Growth performance and bioconversion efficiency of BSF larvae fed different SMS substitution levels (0–100%)

Parameters	SMS substitution (%)					
	0	20	40	60	80	100
Total amount of feed (g DM)	150.00 ± 0.00^a^	150.00 ± 0.00^a^	150.00 ± 0.00^a^	150.00 ± 0.00^a^	150.00 ± 0.00^a^	150.00 ± 0.00^a^
Survival rate (%)	97.25 ± 0.75^a^	98.75 ± 1.30^a^	99.25 ± 1.03^ab^	99.75 ± 0.43^ab^	99.00 ± 1.17^ab^	96.00 ± 1.62^ac^
Individual larval weight (mg FM)	205.39 ± 2.22^a^	229.08 ± 4.65^b^	178.34 ± 2.35^c^	115.43 ± 6.91^d^	59.21 ± 7.02^e^	14.06 ± 0.72^f^
Number of prepupae and pupae (%)	0.00 ± 0.00^a^	4.75 ± 2.86^a^	27.00 ± 14.30^b^	6.50 ± 3.57^a^	0.00 ± 0.00^a^	0.00 ± 0.00^a^
Total harvested biomass						
(g FM)	39.95 ± 0.47^a^	45.25 ± 1.25^b^	35.40 ± 0.79^c^	23.03 ± 1.38^d^	11.48 ± 1.13^e^	2.70 ± 0.15^f^
(g DM)	14.29 ± 0.22^a^	17.59 ± 0.44^b^	13.36 ± 0.58^a^	8.14 ± 0.68^c^	2.89 ± 0.40^d^	0.66 ± 0.03^e^
Frass residue (g DM)	122.68 ± 1.20^a^	80.37 ± 2.22^b^	83.78 ± 0.59^c^	99.67 ± 0.67^d^	128.91 ± 0.83^e^	145.22 ± 1.00^f^
Larval growth rate (LGR; g FM/d)	3.87 ± 0.05^a^	4.41 ± 0.13^b^	3.42 ± 0.08^c^	2.18 ± 0.14^d^	1.03 ± 0.11^e^	0.15 ± 0.02^f^
Feed conversion ratio (FCR)	3.76 ± 0.05^a^	3.32 ± 0.09^b^	4.24 ± 0.09^c^	6.54 ± 0.41^d^	13.21 ± 1.39^e^	55.72 ± 3.00^f^
Efficiency of conversion of the ingested feed (ECI)	0.52 ± 0.02^a^	0.26 ± 0.01^b^	0.20 ± 0.01^c^	0.16 ± 0.01^d^	0.14 ± 0.02^d^	0.15 ± 0.04^bcd^
Substrate reduction (%)	18.21 ± 0.80^a^	46.42 ± 1.48^b^	44.15 ± 0.39^c^	33.56 ± 0.45^d^	14.06 ± 0.56^e^	3.18 ± 0.67^f^
Waste reduction index (WRI; g DM/d)	2.73 ± 0.12^a^	6.96 ± 0.22^b^	6.62 ± 0.06^c^	5.03 ± 0.07^d^	2.11 ± 0.08^e^	0.48 ± 0.10^f^

Data represent means ± SD (*n* = 200). Different letters (a–f) within a row indicate statistically significant differences between groups (*P* < 0.05; one-way ANOVA or Welch’s ANOVA)

Ten days after starting the experiment, no prepupae or pupae were detected in populations provided pure CF and 80–100% SMS, while some larvae (≤ 6.5%) had already developed into prepupae in groups fed with 20% and 60% SMS. Here, the number of prepupae varied between 1–10/box. Feeding 40% SMS resulted in a significant acceleration of development, since 27% of the larvae (9–42 larvae/box) had already reached the prepupal stage on the day of harvest (*F*_5,18_ = 8.78; *P* < 0.008). The number of emerging prepupae was not correlated with the SMS level (*r* = − 0.15; *P* = 0.49). Both fresh matter and dry matter of the total harvested biomass were highest in the 20% SMS group at 43.3–46.6 g FM (*F*_5,18_ = 907.75; *P* ≤ 0.00003) and 17.0–18.2 g DM (*F*_5,18_ = 672.44; *P* < 0.00001), respectively, and exceeded the yield of the CF group by 13.3–23.1% (Table 1). The total harvested biomass was strongly negatively correlated with increasing SMS level and decreased by up to 94.0% FM (*r* = − 0.95; *P* < 0.00001) and 96.2% DM (*r* = − 0.93; *P* < 0.00001) when compared to the CF and 20% SMS groups, respectively. Interestingly, the frass residue in groups fed 20% and 40% SMS was substantially reduced by 34.5% and 31.7% respectively compared to the CF group (*F*_5,18_ = 1396.15; *P* < 0.00001). The amount of frass residue was positively correlated with increasing SMS level (*r* = 0.97; *P* < 0.00001), whereby feeding 100% SMS resulted in a difference of only 4.8 g DM between the weighed frass residue and the feed initially supplied. With 4.2–4.6 g FM/d, the larval growth rate (LGR) was highest in the 20% SMS treatment and exceeded the CF control by 14% (*F*_5,18_ = 890.35; *P* = 0.00003). In general, the LGR decreased sharply with an increase in SMS level (*r* = − 0.99; *P* < 0.00001). When feeding pure SMS, reductions of 96.1% and 96.6% were observed compared to CF and 20% SMS, respectively (*F*_5,18_ = 890.35; *P* < 0.00001).

Besides the biomass yield and larval development, parameters such as utilization efficiency and waste reduction also contribute to circular economy. The 20% SMS substitution improved the feed conversion ratio (FCR) by 11.7% (3.2–3.5) in comparison to pure CF (*F*_5,7.8_ = 197.13; *P* = 0.009). FCR correlated positively with increasing SMS level (*r* = 0.81; *P* = 0.00002) and reached values up to 14.8- and 16.8-fold higher than in the CF and 20% SMS groups, respectively, reflecting the low growth rates and high frass residues reported above. Larvae of all substitution groups were found to have a significantly ≥ 50% lower efficiency of conversion of the ingested feed (ECI) than those provided pure CF (*F*_5,8.0_ = 167.67; *P* ≤ 0.0003). Here, ECI was negatively correlated with increasing SMS level (*r* = − 0.83; *P* = 0.00001). The 20–60% SMS groups showed 1.8–2.5-fold higher substrate reductions compared to their CF counterparts (*F*_5,18_ = 1398.30; *P* < 0.00001), wherein feeding 20% SMS led to the greatest reduction (Table 1). The same was also noted for the waste reduction index (WRI). In fact, the 20–60% SMS groups achieved 1.8–2.5-fold higher daily reduction values than the CF group (*F*_5,18_ = 1403.29; *P* < 0.00001), with 20% SMS resulting in the greatest WRI. Both substrate reduction and WRI were negatively correlated with increasing SMS level (*r* = − 0.97; *P* < 0.00001).

### Optimization of stocking density

While the majority of parameters recorded were significantly improved by substituting 20% SMS, it became apparent that the values decreased markedly in the 40–60% SMS groups (Table 1). Therefore, it was decided to consider a maximum replacement of 50% for further trials. As several diets were not fully digested by the larvae at a density of 200 individuals/box, the amount of feed supplied was reduced from 150 to 100 g DM and variations in stocking density (200–1000 larvae/box) were tested.

**Fig. 1:**
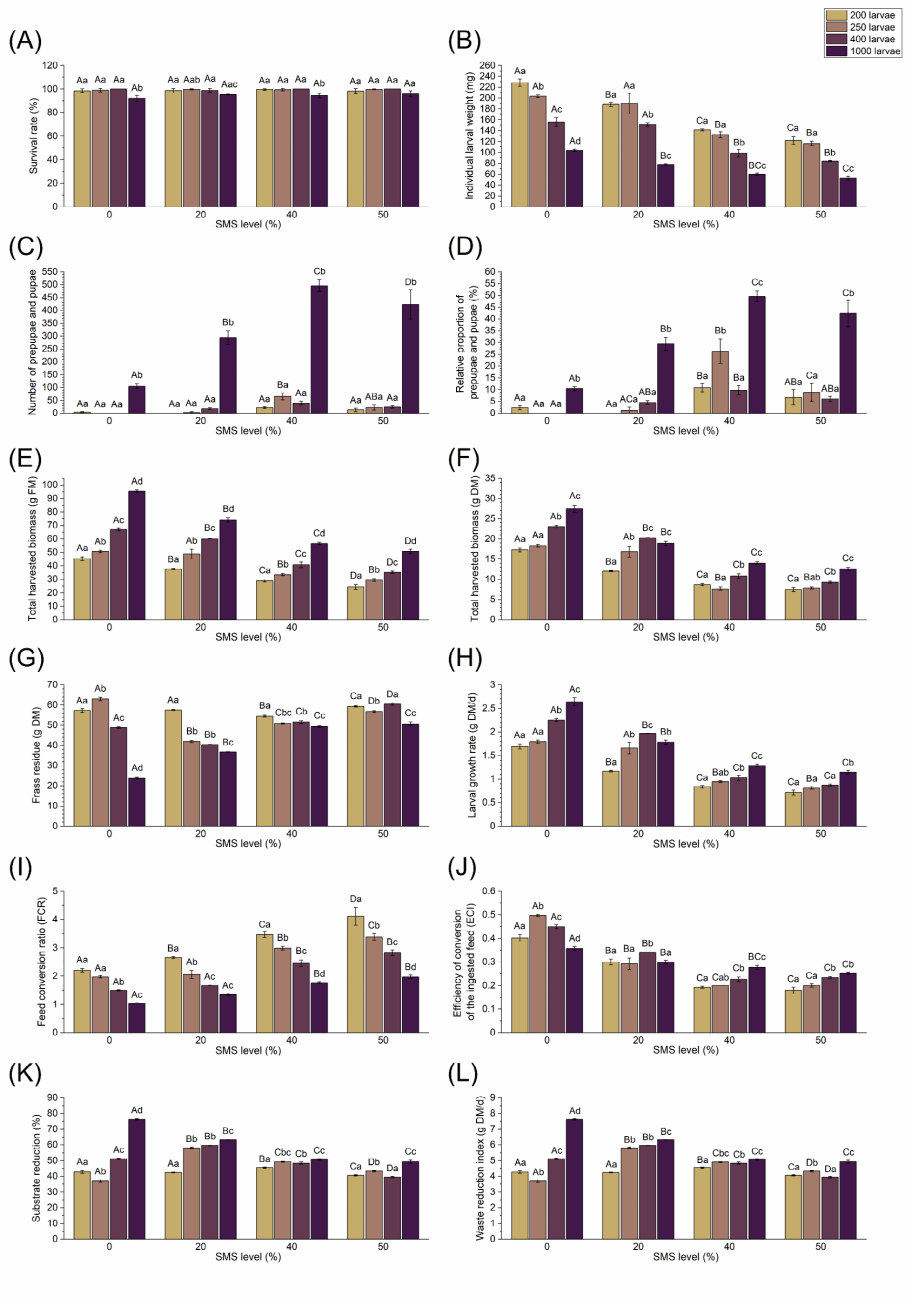
Performance and bioconversion of BSF larvae under various densities (200–1000 larvae/box) and SMS substitutions (0–50%). Data represent means ± SD. (**A**) survival rate, (**B**) individual larval weight, number (**C**) and relative proportion (**D**) of prepupae and pupae, total harvested biomass in FM (**E**) and DM (**F**), frass residue (**G**), larval growth rate (**H**), feed conversion ratio (**I**) and efficiency of conversion (**J**), substrate reduction (**K**) and waste reduction index (**L**). Different letters indicate statistically significant differences, with lowercase letters (a–d) referring to data within an SMS group and uppercase letters (A–D) referring to data within a density group (*P* < 0.05; two-way ANOVA)

All groups received exactly the same amount of 100 g DM of the corresponding diet. In general, survival rate of all groups was high with 92–100%. We found statistically significant differences in the average survival rate for the density (*F*_3_ = 31.38; *P* < 0.00001), but not for the SMS level (*F*_3_ = 1.29; *P* = 0.29). No significant interaction between these terms was calculated (*F*_9_ = 1.17; *P* = 0.34). A Tukey post-hoc test revealed that the highest density reduced the survival rate significantly compared to lower density groups when fed with 0% (*P* ≤ 0.0003) or 40% (*P* ≤ 0.00003) SMS diets (Fig. 1A). Individual weight differed significantly for the density (*F*_3_ = 527.78; *P* < 0.00001) and the SMS level (*F*_3_ = 347.00; *P* < 0.00001), wherein interactions of both variables were verified (*F*_9_ = 10.81; *P* < 0.00001). The highest individual larval weight (220–239 mg FM) was obtained at 200 larvae/box in the 0% SMS group, which was ≥ 16.8% greater than in all SMS containing diets. In general, the weight of the larvae decreased successively with increasing SMS level and density. Feeding 50% SMS at 1000 larvae/box resulted in a weight reduction of 50.3% and 77.1% (*P* < 0.00001) compared to the highest and lowest density within the CF control, respectively (Fig. 1B). Besides the 0% SMS group, individual weight was highest at 200–250 larvae/box in all diets containing SMS (*P* ≤ 0.0002).

The number of larvae that developed into prepupae or pupae differed significantly for the density (*F*_3_ = 855.72; *P* < 0.00001) and the SMS level (*F*_3_ = 101.40; *P* < 0.00001), and both variables interacted with each other (*F*_9_ = 54.02; *P* < 0.00001). When comparing different densities within the same SMS level, the number of prepupae or pupae counted was significantly higher at 1000 larvae/box than in all other groups (*P* < 0.00001). At 200–400 larvae/box, SMS substitution had a minor effect on the appearance of prepupae and pupae, whereas their quantity increased with rising SMS level at 1000 larvae/box, reaching a maximum of 496 at 40% SMS (Fig. 1C). The same was observed for the relative proportion of prepupae or pupae, which was significantly affected by the density (*F*_3_ = 285.59; *P* < 0.00001), the SMS level (*F*_3_ = 124.27; *P* < 0.00001), and their interactions (*F*_3_ = 19.76; *P* < 0.00001). In general, highest proportions for all densities were found at 40% SMS. In the 200–400 larvae/box densities, prepupae or pupae formation varied between the SMS levels in a non-linear pattern. Feeding 40% SMS led to a significant increase of ≥ 66.2% at 250 larvae/box compared to the other SMS levels (*P* < 0.00001). The proportion of prepupae or pupae was highest at 1000 larvae/box compared to all other densities independent of the SMS level (*P* ≤ 0.04; Fig. 1D).

Fresh and dry matter of the total harvested biomass were highest in the 0% SMS diet, followed by 20% SMS substitution with a mean reduction of 14.7% FM and 20.9% DM, respectively. Both variables were significantly affected by the density (*F*_3_ = 1321.00; *P* < 0.00001 and *F*_3_ = 388.37; *P* < 0.00001), the SMS level (*F*_3_ = 968.80; *P* < 0.00001 and *F*_3_ = 1258.58; *P* < 0.00001), and interactions thereof (*F*_9_ = 41.33; *P* < 0.00001 and *F*_9_ = 31.00; *P* < 0.00001). Here, the harvested biomass in all groups grew between 96.4 and 111.0% FM (*P* < 0.03) and 57.2–68.2% DM with increasing density, whereas higher SMS levels led to a reduction of biomass yield (Fig. 1E, F). As a consequence, biomass decreased by up to 47.4% FM and 59.8% DM when CF was replaced by 50%. However, biomass yield DM did not differ between the highest SMS levels of 40–50% within the same density (*P* ≥ 0.13). We found statistically significant differences in the average frass residue for the density (*F*_3_ = 2042.79; *P* < 0.00001) and the SMS level (*F*_3_ = 1024.58; *P* < 0.00001), wherein interactions of both variables were verified (*F*_9_ = 625.53; *P* < 0.00001). In general, the frass residue decreased while the density increased throughout all diets, particularly in groups fed with 0–20% SMS level. When comparing the lowest and highest densities tested, the amount of frass residue was reduced by 58.4% (*P* < 0.00001) and 36.0% (*P* < 0.00001) for 0% SMS or 20% SMS, respectively (Fig. 1G). With 27.0% difference, the reduction of frass residue at 20% SMS and 250 larvae/box was remarkably higher compared to the lowest density (*P* < 0.00001) and all other diet groups (*P* < 0.00001). An SMS level of ≥ 40% led to higher amounts of frass residue across all densities.

LGR was significantly affected by the density (*F*_3_ = 317.93; *P* < 0.00001) and the SMS level (*F*_3_ = 1326.80; *P* < 0.00001), wherein interactions of both variables were calculated (*F*_9_ = 33.73; *P* < 0.00001). Here, the LGR increased with higher densities and peaked at 1000 larvae/box, whereby the 0% SMS group reached a ≥ 32.4% higher rate than their counterparts (*P* < 0.00001). However, LGR of the 20% SMS group did not differ significantly between 250 and 1000 larvae/box (*P* = 0.22), while 400 larvae/box peaked and exceeded both by 16.0% and 9.7%, respectively (*P* ≤ 0.01). We observed that the LGR decreased with increasing SMS level, although it did not differ between the highest SMS levels of 40–50% within the same densities (*P* ≥ 0.09; Fig. 1H). SMS level (*F*_3_ = 368.10; *P* < 0.00001), density (*F*_3_ = 434.06; *P* < 0.00001), as well as their interactions (*F*_9_ = 9.01; *P* < 0.00001) significantly affected the FCR. The FCR improved with increasing density independent of the diet. For this, the best ratios were obtained in the 0–20% SMS groups at 1000 larvae/box (*P* ≤ 0.004). Importantly, the conversion ratio of 0% and 20% SMS at 250–1000 larvae/box did not differ significantly (*P* ≥ 0.10). In contrast, the FCR of the 40–50% SMS diets increased significantly (*P* ≤ 0.006; Fig. 1I). The ECI was affected by the density (*F*_3_ = 35.17; *P* < 0.00001) and SMS level (*F*_3_ = 1027.76; *P* < 0.00001), whereby interactions of both variables were verified (*F*_9_ = 51.06; *P* < 0.00001). Generally, ECI was highest in the 0% SMS group at 250 larvae/box (*P* ≤ 0.0004). Besides 400 larvae/box (*P* ≤ 0.005), no differences were determined between the densities within the 20% SMS group (*P* > 0.99). No linear effect of the density was determined throughout the diets. Feeding higher SMS substitutions led to a successive decline in ECI, although no further decrease was found for larvae provided with 50% SMS instead of 40% SMS (*P* ≥ 0.21; Fig. 1J).

The proportion of substrate reduced differed significantly for the density (*F*_3_ = 2050.27; *P* < 0.00001) and the SMS level (*F*_3_ = 1028.75; *P* < 0.00001), and both variables interacted with each other (*F*_9_ = 631.30; *P* < 0.00001). Besides the 1000 larvae/box density, no linear effect of substrate reduction with SMS level was observed. As density increased, the substrate reduction improved independently of the diet. The substrate reduction was highest at 1000 larvae/box (*P* ≤ 0.003), with 0% SMS exceeding the other diets by ≥ 17.1% (*P* < 0.00001). It was found that feeding 20% SMS at 250–400 larvae/box resulted in a significantly ≥ 13.4% higher substrate reduction in comparison to all other diets within the same densities (*P* < 0.00001; Fig. 1K). The same pattern was noticed for the WRI. The WRI differed significantly for the density (*F*_3_ = 2089.72; *P* < 0.00001) and the SMS level (*F*_3_ = 1051.88; *P* < 0.00001), and both variables interacted with each other (*F*_9_ = 640.85; *P* < 0.00001). As density increased, WRI improved regardless of the diet, with 1,00 larvae/box being the highest (*P* ≤ 0.004). In general, the WRI was highest for the 0% SMS diet, followed by 20% SMS substitution, where the indices did not differ significantly at 200 larvae/box (*P* > 0.99; Fig. 1L). Feeding 20% SMS at 250–400 larvae/box resulted in a significantly higher WRI than all other diets when comparing the same densities (*P* < 0.00001).

### Nutritional analysis

The highest individual weight of larvae was found at a density of 250 larvae/box within the SMS containing groups. Furthermore, when feeding 20% SMS at 250 larvae/box, the harvested biomass, LGR and FCR did not differ significantly from the 0% SMS control, whereas some of the higher densities led to a decrease. In fact, the frass residue, substrate reduction and WRI were even improved at 250 larvae/box in the 20% SMS group. Therefore, the nutritional composition of the feed, larvae, and frass at a density of 250 larvae/box were examined.

The diets used in this study had a comparably low moisture content of < 20% and varied in their chemical composition (Table 2). Here, the highest crude ash content was found in the 0% SMS diet (*F*_3,8_ = 139.28; *P* ≤ 0.00007). As the SMS proportion increased, the crude fiber content also increased by up to 7.8-fold (*F*_3,4.3_ = 579.74; *P* = 0.00001), whereas the crude fat content decreased by half at 50% SMS compared to pure CF (*F*_3,3.3_ = 11.99; *P* = 0.01). The same applied for the total nitrogen and crude protein contents, which were reduced by ~ 30% when CF was replaced by 50% SMS (*F*_3,8_ = 55.79; *P* < 0.00001 and *F*_3,8_ = 59.70; *P* < 0.00001).

**Table 2:** Chemical composition of the diets substituted with 0–50% SMS

Parameters	SMS substitution (%)			
	0	20	40	50
Moisture (%)	13.5 ± 0.5^a^	16.1 ± 0.0^a^	13.3 ± 0.3^a^	13.2 ± 0.4^a^
Crude ash (%DM)	13.1 ± 0.1^a^	11.1 ± 0.1^b^	10.1 ± 0.3^c^	8.8 ± 0.3^d^
Crude fiber (%DM)	2.4 ± 0.4^a^	9.3 ± 0.3^b^	15.9 ± 1.2^c^	18.7 ± 0.3^c^
Crude fat (%DM)	2.5 ± 0.2^a^	1.9 ± 0.0^ab^	1.2 ± 0.4^ab^	1.2 ± 0.2^b^
Total nitrogen (%DM)	2.7 ± 0.0^a^	2.3 ± 0.0^b^	2.1 ± 0.1^c^	1.9 ± 0.1^c^
Kjeldahl factor	6.51	6.52	6.52	6.43
Crude protein (%DM)	17.3 ± 0.1^a^	15.0 ± 0.3^b^	13.4 ± 0.4^c^	12.0 ± 0.7^d^

Besides the moisture content, analyzed parameters are given as a percentage of DM (%DM). Data represent means ± SD (*n* = 3). Different letters (a–d) within a row indicate statistically significant differences between groups (*P* < 0.05; one-way ANOVA or Welch’s ANOVA)

The different diets fed to the BSF larvae led to significant differences in their chemical composition. Lyophilization reduced the larval moisture content from 67.7 to < 7.8% (Table 3). The crude ash content of larvae provided 50% SMS was 11.1 to 15.5% higher than that of larvae fed pure CF or 20% SMS (*F*_3,8_ = 27.56; *P* < 0.003). The same was observed for the crude fiber content, which increased by 42.0% when CF was replaced by 50% SMS (*F*_3,8_ = 41.15; *P* = 0.00005). The crude fat revealed an opposite pattern, with the lowest content (46.7% reduction) found in the larvae fed the 50% SMS diet (*F*_3,8_ = 91.34; *P* < 0.00001). All SMS containing diets improved the total nitrogen (*F*_3,8_ = 57.21; *P* ≤ 0.02) and, thus, crude protein content (*F*_3,8_ = 58.55; *P* ≤ 0.02) in BSF larvae significantly compared to pure CF, with 40–50% SMS showed the highest values (Table 3).

**Table 3:** Chemical composition of BSF larvae reared on diets substituted with 0–50% SMS

Parameters	SMS substitution (%)			
	0	20	40	50
Moisture (%)^†^	6.4 ± 0.3^a^	6.6 ± 0.8^a^	7.1 ± 0.1^a^	7.8 ± 0.0^b^
Crude ash (%DM)	15.7 ± 0.1^a^	14.9 ± 0.2^a^	17.5 ± 0.4^b^	17.6 ± 0.5^b^
Crude fiber (%DM)	3.4 ± 0.2^a^	4.6 ± 0.2^b^	5.7 ± 0.3^c^	5.8 ± 0.3^c^
Crude fat (%DM)	21.0 ± 0.5^a^	20.8 ± 1.0^a^	14.2 ± 0.9^b^	11.2 ± 0.3^c^
Total nitrogen (%DM)	5.8 ± 0.1^a^	6.3 ± 0.1^b^	7.2 ± 0.1^c^	7.2 ± 0.1^c^
Kjeldahl factor	5.84	5.85	5.86	5.84
Crude protein (%DM)	34.0 ± 0.8^a^	36.9 ± 0.8^b^	42.2 ± 0.5^c^	41.9 ± 0.8^c^

Besides the moisture content, analyzed parameters are given as a percentage of DM (%DM). Data represent means ± SD (*n* = 3). Different letters (a–c) within a row indicate statistically significant differences between groups (*P* < 0.05; one-way ANOVA or Welch’s ANOVA)

^†^After lyophilization

After harvesting the larvae, the frass of the different groups had an average moisture content of 63.2%, which was reduced to < 15.7% by lyophilization (Table 4). The crude ash content of frass obtained from pure CF did not differ significantly from 20 to 40% SMS, while a 50% SMS replacement led to a 24.9% lower content (*F*_3,3.3_ = 66.77; *P* = 0.01). Frass from all SMS containing diets had ≥ 6.6-fold higher crude fiber contents compared to pure CF (*F*_3,8_ = 708.33; *P* < 0.00001). There was no significant difference in the crude fat content of the frass among the groups (*F*_3,3.9_ = 1.72; *P* = 0.30). All SMS containing frass showed significantly lower total nitrogen (*F*_3,8_ = 138.94; *P* ≤ 0.00004) and crude protein contents (*F*_3,8_ = 105.06; *P* ≤ 0.0002) compared to pure CF. There were no significant differences in crude fiber, protein, and total nitrogen contents between the 40–50% SMS frass (Table 4).

**Table 4:** Chemical composition of BSF frass obtained from diets substituted with 0–50% SMS

Parameters	SMS substitution (%)			
	0	20	40	50
Moisture (%)^†^	13.9 ± 1.3^a^	13.3 ± 0.2^ab^	15.6 ± 0.3^a^	15.7 ± 0.1^ac^
Crude ash (%DM)	21.6 ± 0.0^a^	25.7 ± 1.0^ab^	19.7 ± 0.6^ac^	16.2 ± 0.5^d^
Crude fiber (%DM)	3.2 ± 0.4^a^	21.5 ± 0.8^b^	29.9 ± 0.7^c^	30.0 ± 0.7^c^
Crude fat (%DM)	0.8 ± 0.0^a^	0.8 ± 0.1^a^	1.0 ± 0.3^a^	0.9 ± 0.1^a^
Total nitrogen (%DM)	2.5 ± 0.1^a^	2.0 ± 0.0^b^	1.7 ± 0.0^c^	1.7 ± 0.0^c^
Kjeldahl factor	5.64	5.82	5.91	5.97
Crude protein (%DM)	14.0 ± 0.4^a^	11.9 ± 0.2^b^	9.8 ± 0.2^c^	10.3 ± 0.3^c^

Besides the moisture content, analyzed parameters are given as a percentage of DM (%DM). Data represent means ± SD (*n* = 3). Different letters (a–d) within a row indicate statistically significant differences between groups (*P* < 0.05; one-way ANOVA or Welch’s ANOVA)

^†^After lyophilization

**Fig. 2:**
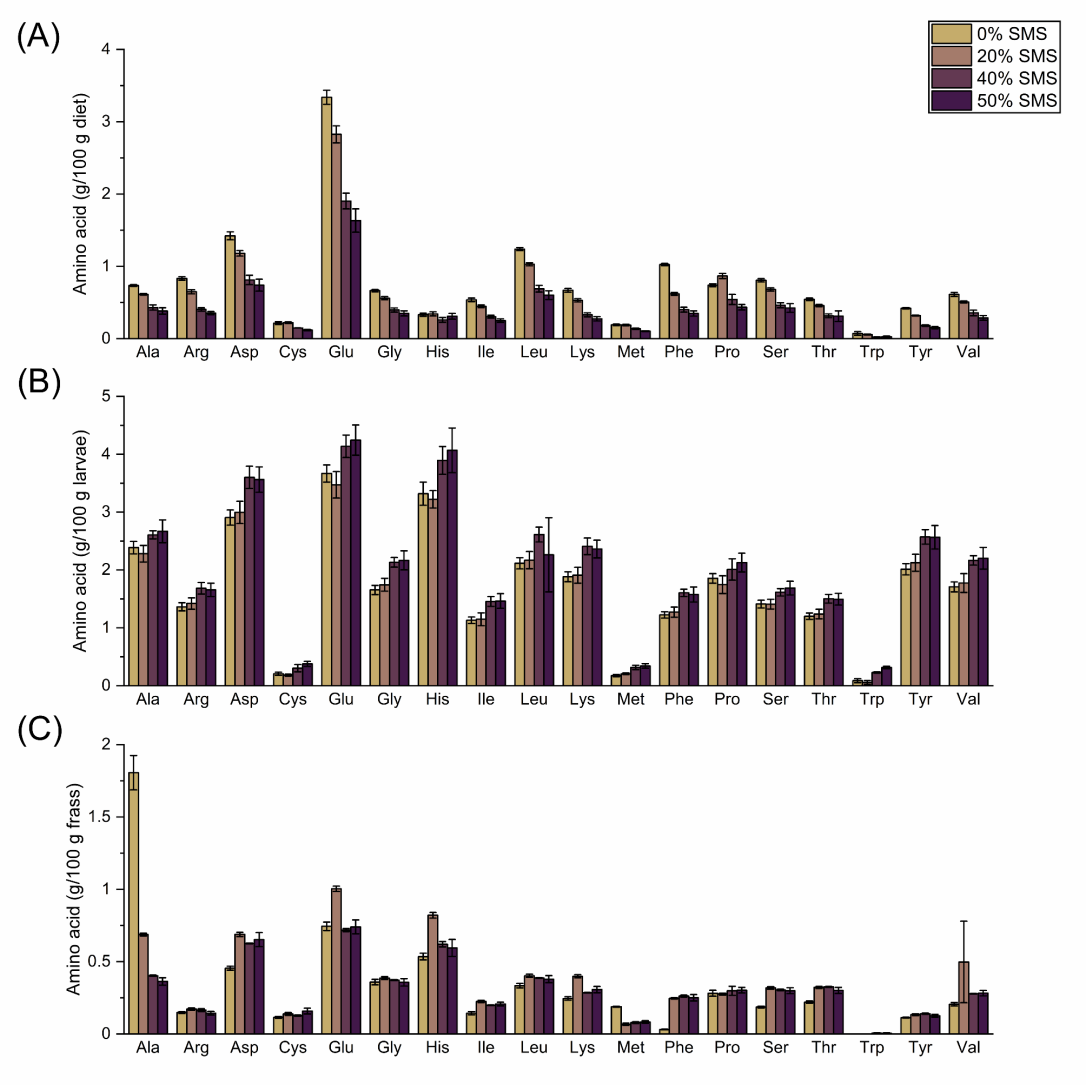
Amino acid composition of (**A**) diets substituted with 0–50% SMS, (**B**) BSF larvae reared on them, and (**C**) the corresponding frass. Data represent means ± SD (*n* = 3) and are given as g/100 g sample

The amino acid content decreased with increasing SMS substitution in the feed, although there was no major difference between 40 and 50% SMS substitution (Fig. 2A). Glutamine was the predominant amino acid in all diets, whereas methionine, cysteine and tryptophan were limiting. In general, the amino acid content of the larvae was markedly higher than that of the feed or the frass and contained great amounts of aspartic acid, glutamine and histidine (Fig. 2A–C). Larvae fed 40–50% SMS achieved higher amino acid contents than their counterparts fed lower SMS levels. The amino acid content of the frass was considerably reduced, and tryptophan was almost completely removed (Fig. 2C).

The dominant FA in the diets was C18:2 (38.6–46.2%), followed by C18:1 and C16:0 (Table 5). Besides the FAs found in the diets, additional ones were detected in the larvae, namely C12:0 (58.3–73.9%), C14:0 (9.7–11.6%), and C16:1 (1.8–3.2%). The frass had the most diverse FA profile, especially in the 40–50% SMS groups. C16:0 (22.6–35.4%) and C18:1 (23.0–34.6%) were the most abundant FAs in all frass groups, followed by C18:2 (10.4–34.7%). Here, we also detected methyl-branched FAs, which are rare to find in nature (Table 5).

**Table 5:** Fatty acid profile of diets substituted with 0–50% SMS, BSF larvae, and the frass

Fatty acids	Substrate				Larvae				Frass			
SMS level (%)	0	20	40	50	0	20	40	50	0	20	40	50
C12:0	–	–	–	–	63.5 ± 1.1	73.9 ± 1.0	59.9 ± 2.3	58.3 ± 1.1	–	–	–	–
C14:0	–	–	–	–	11.6 ± 0.2	9.8 ± 0.2	10.8 ± 0.6	9.7 ± 0.2	–	–	3.5 ± 0.5	2.7 ± 0.6
C14:0*	–	–	–	–	–	–	–	–	–	3.5 ± 0.0	13.5 ± 0.7	6.8 ± 0.2
C15:0*	–	–	–	–	–	–	–	–	–	–	4.0 ± 0.2	2.7 ± 0.3
C16:0*	–	–	–	–	–	–	–	–	–	–	4.7 ± 0.2	2.6 ± 0.4
C16:0	23.6 ± 0.4	24.4 ± 0.8	25.8 ± 0.3	25.7 ± 0.2	12.5 ± 0.4	9.5 ± 0.3	15.4 ± 1.2	15.6 ± 0.4	34.4 ± 0.6	35.4 ± 0.1	28.4 ± 0.2	22.6 ± 0.4
C18:0	2.5 ± 0.1	3.2 ± 0.2	3.3 ± 0.0	3.0 ± 0.0	1.2 ± 0.1	–	1.2 ± 0.1	1.5 ± 0.0	6.2 ± 0.1	7.4 ± 0.0	4.9 ± 0.5	3.4 ± 0.3
Σ saturated fatty acids	26%	28%	29%	29%	89%	93%	87%	85%	41%	46%	59%	41%
C16:1	–	–	–	–	3.2 ± 0.1	1.9 ± 0.1	2.2 ± 0.0	1.8 ± 0.1	–	–	2.1 ± 0.1	3.6 ± 0.1
C18:1	27.7 ± 0.1	29.9 ± 0.4	31.5 ± 0.3	32.7 ± 0.4	6.9 ± 0.4	4.9 ± 0.4	8.0 ± 0.3	9.5 ± 0.4	23.0 ± 0.6	33.6 ± 0.0	28.5 ± 0.4	34.6 ± 0.9
C19:1	–	–	–	–	–	–	–	–	–	1.7 ± 0.0	–	–
Σ monounsaturated fatty acids	28%	30%	32%	33%	10%	7%	10%	11%	23%	35%	31%	38%
C18:2	46.2 ± 0.5	42.5 ± 0.4	39.4 ± 0.6	38.6 ± 0.6	1.1 ± 0.1	–	2.5 ± 0.1	3.6 ± 0.2	34.7 ± 0.1	17.1 ± 0.0	10.4 ± 0.2	21.0 ± 0.4
C18:3	–	–	–	–	–	–	–	–	1.7 ± 0.1	1.3 ± 0.0	–	–
Σ polyunsaturated fatty acids	46%	42%	39%	38%	1%	0%	3%	4%	36%	19%	10%	21%

Data represent means ± SD (*n* = 3) and are given as a percentage of total fatty acids (*methyl branched fatty acids;– not detected or sum of less than 1%)

## Discussion

Spent mushroom substrate, also known as spent mushroom compost, mushroom bran, mushroom residue, or mushroom soil (Oei et al. 2007) contains around 80% of unused nutrients (Moon et al. 2012), making them a promising feed ingredient (Bapista et al. 2023; Foluke et al. 2014). However, there has been limited research into the use of SMS as feed for insects (Kim et al. 2014; Li et al. 2020, 2021; Mao et al. 2023). This study evaluated the potential of using *Pleurotus eryngii* and *Lentinula edodes* SMS as one of the rearing substrates for BSF larvae. Both species belong to the highest-produced mushrooms in terms of yield (Zhang et al. 2015) and industrialization (Li 2018), indicating large quantities of corresponding SMS. Other SMS that fall into these categories originate from *Agaricus bisporus* and *A. arvensis* (Bapista et al. 2023; Girotto and Piazza 2022), *Auricularia cornea*, *A. auricularia*, and *A. heimuer* (Du et al. 2022; Wei et al. 2020; Li et al. 2021), *Pleurotus citrinopileatus* and *P. ostreatus* (Li et al. 2020, 2021), and *Flammulina velutipes* (Kim et al. 2014) production. Although the composition of SMS varies, they mainly contain around 40% cellulose, 28% hemicellulose, and 32% lignin. Thus, like our study, others usually tested a gradual substitution of standard feed with SMS. In this work, we gradually replaced chicken feed with an SMS mixture. Similarly, gradual substitutions of wheat and rice bran with 0–70% *L. edodes* SMS (Li et al. 2020) and wheat bran with *Pleurotus eryngii* and *F. velutipes* SMS (Kim et al. 2014) were carried out for feeding *Tenebrio molitor* (Coleoptera: Tenebrionidae) larvae. Li et al. (2021) conducted feeding experiments for BSF larvae with *L. edodes* SMS and food waste. It is to be noted that food waste is not allowed as feed in the EU (Regulation (EC) No 999/2001). Another study fed SMS from not specified mushrooms and distiller’s grain to BSF larvae (Mao et al. 2023).

The survival rates of BSF larvae in the current substitution experiment were between 96 and 99.8%. Similarly, the survival of BSF larvae was not significantly different when fed with Gainesville diet (control consisting of alfalfa, wheat bran, and corn meal) or SMS of *A. heimuer*, *L. edodes*, *P. eryngii*, or *P. citrinopileatus* (Li et al. 2021). However, Li et al. (2020), postulated that all *T. molitor* larvae died when fed with 100% of *A. auricularia*, *P. ostreatus*, or *P. citrinopileatus* SMS, whereas survival for *L. edodes* and *P. eryngii* SMS were 37% and 1.3%, respectively. The substitution of wheat bran and rice bran with *L. edodes* SMS resulted in > 90% survival, with the lowest rate (~ 92%) observed at 70% substitution of wheat bran. These results highlight the potential of rearing insects, particularly BSF larvae, on SMS. Some mushrooms may produce secondary metabolites that might affect the insect’s performance or, in some cases, lead to a lower survival. This is not the factor that influenced our study, as the survival rate was high even at 100% SMS level (Fig. 1A).

However, we found differences between the individual larval weights when chicken feed was substituted with SMS. The highest individual larval weight (223–234 mg FM) was not in the control diet but with 20% SMS substitution. As the SMS level increased, the individual larval weight decreased, which might be related to a reduction of nutritional value (Table 2). In the stocking density trials, the individual weight was significantly different both in terms of SMS level and larval density. The reduction in individual weight with increasing density might be because of the growing competition between the larvae. Similarly, the individual weight of *T. molitor* larvae reduced as the proportion of *P. eryngii* SMS increased in the diet (Kim et al. 2014). After 30 d, the weight of *T. molitor* larvae on pure *L. edodes* (0.5 mg) and *P. eryngii* (negligible to be measured) SMS were comparatively lower than those reared on the control diets (3.6–6.8 mg). *T. molitor* larvae weighed 4.8 mg on 60:40 SMS: wheat bran and 4.6 mg on 60:40 SMS: rice bran diet. The authors hypothesize that pure SMS could not meet the nutritional requirements of the larvae, which might be due to its physical, chemical, and biological properties (Li et al. 2020). Feeding mixtures therefore appear to compensate for such deficiencies. Cai et al. (2017) prepared a mixture of wheat bran or kitchen waste and root waste from *F. velutipes* as feed for BSF larvae. In our study, the mushroom stems attached to the SMS blocks were removed to maintain uniformity. However, using the SMS blocks with mushroom stems would provide additional nutrients. The larval weight on 50:50 *L. edodes*: *P. eryngii* SMS in the current study was 14 mg (Table 1). According to Li et al. (2021), BSF larvae weighed 69 mg and 38 mg on pure *L. edodes* or *P. eryngii* SMS, respectively. The substitution of food waste with *L. edodes* SMS resulted in larval weights between 160 and 242 mg (Li et al. 2021). When substituting similar levels of CF, we yielded larval weights ranging between 59 and 229 mg. The reason for this difference in range could be the feeding rate. In this work, 150 g DM feed/200 larvae were used, while Li et al. (2021) provided three times more with 90 g DM feed/40 larvae. Interestingly, the highest weights in both studies were not in the control diet. In fact, the highest larval weights were obtained at 60% SMS with 260 mg (Li et al. 2021) and at 20% SMS with 229 mg, exceeding the corresponding controls by ~ 11%.

The number of prepupae and pupae formed was highest at 40% SMS, while none were found in the 100% SMS diet (Table 1; Fig. 1D). Similarly, no prepupae were detected when pure *L. edodes* and *P. eryngii* SMS were fed (Li et al. 2021). Here, a density of 1000 larvae accelerated the prepupae or pupae formation compared to lower densities. However, the prepupae and pupae were of smaller size. Presumably, the lower nutrient availability in SMS caused them to develop faster, although individuals were smaller, to reach the adult stage despite unfavourable conditions. Another possible reason could be higher temperatures in the rearing boxes due to increased larval interactions at 1000 larvae density, leading to faster development. For *T. molitor*, a diet of 40:60 wheat bran: *L. edodes* SMS also resulted in smaller pupae compared to those reared on pure wheat bran or a lower substitution of SMS (Li et al. 2020). According to Li et al. (2021), 32–55% of BSF developed to prepupae on food waste replaced with 20–40% of *L. edodes* SMS. In the substitution experiment, BSF prepupae proportion was 4.8 and 27.0% on CF replaced with 20 or 40% SMS, respectively. Astonishingly, the food waste diet promoted a faster development than the CF diet.

The total harvested biomass in the substitution experiment was 2.7 g FM at 100% SMS and 40.0 g FM for pure CF. Mao et al. (2023) inoculated 0.2 g of BSF larvae on wet distiller’s grain and a not specified SMS-based diet. Here, total harvested larvae biomass was ~ 2.4 and 22.8 g FM for 100% SMS and pure wet distiller’s grain, respectively. Since neither the number of inoculated larvae nor their density is given, a comparison with our results is difficult. All other publications that used SMS to feed insects have not addressed the total harvested biomass, giving no path for comparison.

Frass residue in the substitution experiment was highest when larvae were fed with 100% SMS. Mao et al. (2023) obtained similar results, indicating that the highest frass residues occur with a 100% SMS diet compared to standard diets or mixtures thereof. The authors conclude that the lower nutritional content in SMS is the cause for the lower utilization by the larvae. Furthermore, indigestible components such as fibers, particularly lignocelluloses present in plant-based substrates, are also likely to limit utilization by the larvae (Klüber et al. 2022; Tables 2 and 4).

In the current study, the LGR was highest (4.2–4.6 g FM/d) in the 20% SMS substitutions, while pure SMS resulted in an LGR of 0.2 g FM/d. Similarly, feeding fiber-rich diets such as olive pomace and silage grass, led to a comparable low LGR of 0.2 and 0.7 g FM/d (Veldkamp et al. 2021). Like LGR, FCR was best (3.3) in the 20% SMS diet, whereas higher SMS substitutions caused higher FCRs. According to Oonincx et al. (2015) the FCR was best (~ 1.4) for a high protein-high fat diet and lowest for a low protein-low fat diet (~ 2.6). With 26% crude fiber content, apple pulp also had a higher FCR (8.9) than the CF control (2.1) and a 74.2% lower individual larval weight (Broeckx et al. 2021). This justifies, that such by-products cannot fully meet the requirements for larval growth without mixing or pretreating them (Klüber et al. 2022). Substituting CF with SMS resulted in lower ECI (Fig. 1J; Table 1). Mao et al. (2023) also found an ECI of 24.7% for the 75:25 wheat distiller’s grain: SMS diet and of 2.2% for pure SMS diet. Similar to our diets containing high levels of SMS, ECI was 0.26 for olive pomace and 0.14 for silage grass (Veldkamp et al. 2021), indicating the difficulty of the conversion of fiber-rich feed into biomass by BSF larvae (Li et al. 2015).

The substrate reduction was highest (46.4%) on the 20% SMS diet and lowest (3.2%) on the 100% SMS diet. Substrate reduction of 64.3% and 33.11% was obtained for pure distiller’s grain and pure SMS, respectively (Mao et al. 2023). The higher substrate moisture (10%) and longer feeding period (5 d longer) in the latter study might have stimulated a better decomposition of the feed. Cai et al. (2017) postulated a substrate reduction of 42.3% when mushroom root waste was fed to BSF larvae. In accordance with our data, substrate reduction has been shown to reduce gradually as wheat bran was replaced by olive pomace (Ramzy et al. 2022).

The waste reduction index was highest (7 g DM/d) at 20% SMS substitution, while pure CF and pure SMS had 2.7 and 0.5 g DM/d, respectively. Higher SMS substitutions generally resulted in lower WRI. Cai et al. (2017) observed a similar tendency with a WRI of 2.4 g DM/d for Gainesville diet (control) and 1.6 g DM/d for mushroom root waste. The difference between the WRI of pure SMS and mushroom root waste may be due to the fact that the latter one has a total amino acid content of 6.1 g/100 g DM, which is about twice as high as SMS with 3.1 g/100 g DM (data not shown). In contrast, olive pomace had an identical WRI as pure SMS (Veldkamp et al. 2021).

We observed that the substrates were not fully utilized by the larvae at a density of 200 larvae/box. Hence, higher densities were considered to optimize the substrate utilization (200–1000 larvae per 100 g DM feed). Several densities have already been tested for various substrates, as discussed by Nayak et al. (2024). Although higher larval densities showcase intraspecific competition for resources, there could be beneficial effects due to higher bacterial accumulation and hence associated availability of nutrients (Barragan-Fonseca et al. 2018). Similar to our data, the individual larval weight was shown to decrease as the stocking density increase (Barragan-Fonseca et al. 2018). However, Lopes et al. (2023) postulate that increasing larval density up to 6.3 larvae/cm^2^, increased bioconversion efficiency and larval yield. We also observed an increase of larval yield when the density increased, whereas the efficiency of conversion increased, remained the same or decreased depending on the SMS level (Fig. 1F, J). Yakti et al. (2022) also highlight the differences in larval growth and chemical composition due to changes in larval density.

Besides the larval performance, it is of great interest to perform chemical analyses in order to identify suitable by-products, develop new feeding concepts, evaluate the quality of the products, and suggest appropriate uses for them. With 13.1% DM, the crude ash content of pure CF was highest among all diets tested and reduced gradually as the SMS level increased. Other studies report ash contents between 4 and 6% DM for vegetable, grain, and press cake diets fed to BSF (Bonelli et al. 2020; Galassi et al. 2021; Schreven et al. 2020; Broeckx et al. 2021). Lower ash contents of 0.4–0.9% DM were found for sweet potato, spent coffee, and dough-based diets (Romano et al. 2022). It is not yet clear to what extent ash content influences the larval development. As discussed above, fiber-rich by-products have a high proportion of indigestible components for BSF larvae. In combination with low protein and fat content, this can severely restrict larval growth. These by-products can be improved through a sensible combination with other substrates or a targeted fermentative pretreatment, as shown by Klüber et al. (2022). Li et al. (2021) did not provide the feed composition but the larval crude ash content in the 70:30 food waste: *L. edodes* SMS diet was similar to the 0% SMS diet of the current study. The larval crude fiber content is not addressed in the majority of publications. With 17.8% DM, Li et al. (2021) determined a crude fiber content that was > 3-fold higher than in our larvae. This is probably due to feed residues that were present in the larval gut at the time of harvest. The larval crude protein content increased from 34% DM at 0% SMS to 42% DM at 50% SMS substitution, which is similar to or slightly lower than the literature data. Here, the crude protein content of the diets seems to have a considerable influence. For example, Oonincx et al. 2015 postulated comparable larval crude protein contents of 38–46% DM when feeding diets with 13–23% DM protein (see Table 2), while larvae reared on 30–39% DM protein diets had around 50% DM crude protein (Galassi et al. 2021). In general, however, it should be mentioned that the overwhelming majority of studies state a crude protein content that is too high, as they assume a conversion factor of 6.25. We, on the other hand, worked with a corrected Kjeldahl factor, which was calculated based on the amino acid profile and is 5.85 on average. The amino acid profile of the 50% SMS diet in the current study is similar to that of mushroom root waste, but no information on the larval composition is provided (Cai et al. 2017). Histidine, glutamic acid, cysteine, aspartic acid, alanine, and tyrosine contents of our BSF larvae were comparable or slightly higher than larvae reared on sweet potato and spent coffee (Romano et al. 2022).

The crude fat content of the larvae reared on chicken feed was 26% DM (Galassi et al. 2021). In comparison, Li et al. (2021) found a relatively lower crude fat content of 17.8% DM in larvae fed a 70:30 food waste: *L. edodes* SMS diet. According to Barrgan-Fonseca et al. (2018), the larval fat content varies greater than their protein content depending on the diet. FAs identified from the feed used in the current study had a chain length of C16–C18, including monounsaturated and polyunsaturated ones (Table 5). The frass was found to have a higher variety of FAs, some of them were also methyl-branched, which are extremely rare in nature (Hochmuth and Piel 2009). The substrate’s composition, along with the presence of distinct microbial communities within the larval gut that aid in FA metabolism, or the potential interplay between these factors, may contribute to the observed higher variety of FAs, including methyl-branched ones, in the frass. In particular in larvae, C12:0 and C14:0 proportions were higher than those reported in other studies (St-Hilaire et al. 2007; Romano et al. 2022; Galassi et al. 2021).

The chemical composition of the frass shows the overall utilization of the nutrients from the feed. Here, crude fiber content increased in the frass, while crude protein and fat contents decreased. As suggested by Cai et al. (2017), BSF frass from mushroom by-products could be a promising organic fertilizer.

Commercial BSF producers predominantly use chicken or pig feed as diet for larval fattening, which is neither sustainable nor cost-efficient. *L. edodes* and *P. eryngii* SMS are generated in large quantities during the production of mushrooms and can help to reduce the amount of such feedstuffs by partially replacing them. At the same time, large quantities of these organic by-products could be returned to the food and feed chain and thus contribute to circular bioeconomy efforts. Further research should be conducted on the use of SMS from other mushrooms, combinations thereof, and mixtures with different by-products as feed for insect rearing. Besides, feeding fresh SMS (including mushroom stems) immediately after harvesting could be promising.

## Data Availability

All data and materials are included in the manuscript.

## Abbreviations

ANOVA: Analysis of variance
BSF: Black soldier fly
CF: Chicken feed
DM: Dry matter
ECI: Efficiency of conversion of ingested feed
EU: European Union
FA: Fatty acid
FAME: Fatty acid methyl esters
FCR: Feed conversion ratio
FM: Fresh matter
LGR: Larval growth rate
SMS: Spent mushroom substrates
SR: Substrate reduction
WRI: Waste reduction index
